# *Halobacteria* Formula Improvement of Skin Care—A Randomized, Double‐Blind, Placebo‐Controlled Clinical Study

**DOI:** 10.1111/jocd.16648

**Published:** 2024-11-01

**Authors:** Leong‐Perng Chan, Cheng Da‐Long, Ya‐Ping Tseng, Chia‐Hua Liang

**Affiliations:** ^1^ Department of Otorhinolaryngology‐Head and Neck Surgery, Kaohsiung Municipal ta‐Tung Hospital Kaohsiung Medical University Kaohsiung Taiwan; ^2^ Department of Otorhinolaryngology‐Head and Neck Surgery, Faculty of Medicine, College of Medicine, Kaohsiung Medical University Hospital Kaohsiung Medical University Kaohsiung Taiwan; ^3^ Department of Computer and Communication Shu‐Te University Kaohsiung Taiwan; ^4^ Institute of Basic Medical Sciences National Cheng Kung University Tainan Taiwan; ^5^ Department of Cosmetic Science and Institute of Cosmetic Science Chia Nan University of Pharmacy and Science Tainan Taiwan

**Keywords:** anti‐aging, halobacteria, serum, skin

## Abstract

**Background:**

*Halobacteria trueperi*, an extremophilic microorganism thriving in high‐salt environments, produces extracellular polysaccharides with potential anti‐inflammatory and anti‐aging properties. However, its clinical efficacy in skin improvement remains unclear. This study focuses on *H. trueperi* TCI66207, isolated from the Pacific Ocean at a depth of 662 m near Hualien, and its potential to enhance skin parameters, aiming to develop a novel functional formulation for pharmaceutical and cosmetic use.

**Aims:**

This sudy aims to evaluate the clinical efficacy of *H. trueperi* TCI66207 on various skin parameters and its potential for developing new functional cosmetic formulations.

**Patients/Methods:**

A total of 40 subjects were recruited and randomly divided into two groups: the test group applied a serum containing *H. trueperi* TCI66207, while the placebo group used a basic serum. Subjects were instructed to apply the serum twice daily for 4 weeks. Skin parameters, including moisture, brightness, elasticity, pigmentation (spots and UV spots), texture, wrinkles, pores, and collagen density, were assessed before and after the 4‐week application period.

**Results:**

After 4 weeks of using the *H. trueperi* TCI66207 serum, significant improvements were observed in all measured skin parameters compared to baseline, with notable enhancements in moisture, brightness, elasticity, texture, and collagen density, along with reductions in wrinkles, spots, and pore size.

**Conclusions:**

*Halobacteria trueperi* TCI66207 serum demonstrates a clear ability to improve skin conditions and delay signs of aging, making it a promising candidate for the development of new cosmetic formulations with potent anti‐aging and skin‐rejuvenating properties.

## 1 Introduction

The average age of the world's population is increasing year by year, especially in the population of developed countries [1]. Aging is a natural process. The main feature of aging is the appearance of wrinkles or sagging skin [2]. Skin aging is related to physiological diseases, and it was also shown that the ingredients or methods of use are not suitable for cosmetics. Studies had shown that when the protective barrier function of the skin is disturbed, it can cause dry skin and increase the risk of skin diseases [3]. In recent years, most skin care products had begun to advocate natural ingredient sources to replace chemically processed cosmetics. Based on the above reasons, it was necessary to develop a full range of biotech cosmetics with anti‐wrinkle, anti‐aging, and moisturizing functions.

Marine microorganisms (e.g., fungi and bacteria) are one of the attractive study directions in contemporary science, given that their produced bioactive compounds (e.g., carotenoids, polysaccharides, phenols, etc.) had been identified as potent pharmaceutical and cosmetic agents due to the anti‐aging, anti‐oxidative, anti‐cancer, and anti‐microbial properties [4, 5]. Among them, *Halobacteria* are an intriguing candidate but still at the early exploration phase for the cosmetic applications [6]. In *Halobacteria*, extremely halophilic microbes in the highly saline environment, compatible solutes (osmolytes) are transported out of cytoplasm in response to the rise of extracellular osmolarity [7]. For example, halotolerant and moderate halophilic (3%–15% NaCl) bacteria, such as *H. trueperi* and *Halorhodospira halochloris*, can exploit sugars (e.g., trehalose and sucrose), alcohols, amino acids (e.g., glycine and proline), betaines, ectoines, or their derivatives to adjust the osmotic pressure [8, 9]. Previous studies found that the production of carotenoid compounds (e.g., astaxanthin) in *H. trueperi* was capable of repairing DNA damage in the presence of UV irradiation and chelate iron from the milieu [9]. Moreover, *H. trueperi* can produce exopolysaccharide (EPS), which is beneficial for anti‐aging and anti‐inflammatory activities [10, 11]. However, there are few clinical studies on the skin of *H. trueperi*, and they are worth studying.

In this study, the current explorations of *H. trueperi* with respect to practical applications are in the early stage. Herein, we isolated *H. trueperi* TCI66207 from the 662 m deep Pacific Ocean in Hualien (Taiwan) to develop skin serum and evaluated its anti‐aging effect.

## 2 Materials and Methods

### 2.1 The Manufacturing Process of *H. trueperi* TCI66207

The yeast peptone, glucose, MgSO_4_·7H_2_O, NaCl, KH_2_PO_4_, K_2_HPO_4_, manganese gluconate, and water were used for mixing and fermentation. After fermentation, *H. trueperi* TCI66207 was added, and then the fermentation was carried out. After the fermentation was terminated, a sample was taken for confirmation. After centrifugation, the bacterium mud was collected, water was added to mix, the bacteria were heated, and the supernatant was collected by centrifugation, filled, and sterilized. Then, 5% butanediol, 0.6% hydroxyacetophenone, and 0.6% hexanediol were added to mix, labeled, and stored at 4°C.

### 2.2 Clinical Design

This clinical research was approved by the ethics committee of the Antai Medical Care Corporation Antai Tian‐Sheng Memorial Hospital (TSMH‐IRB 18‐148‐A). All methods were performed following the Declaration of Helsinki and the International Conference on Harmonization (ICH) guidelines on Good Clinical Practice (GCP). Forty adult subjects (mean age of 45.3) were recruited in this trial between January 2019 and January 2020, and informed consent was obtained from all the subjects before the study. The study was performed at the Department of Cosmetic Science and Institute of Cosmetic Science, Chia Nan University of Pharmacy and Science, Taiwan. The 40 eligible subjects were allocated to the experiment group (*H. trueperi* TCI66207 serum; 14 females and six males; mean age of 49.1 years) and placebo group (basic serum; 14 females and four males; mean age of 41.5 years) by block randomization (Table 1). Each subject was informed to apply it twice a day (3–5 mg/cm^2^ once), after washing their face in the morning and after washing their face in the evening, for 4 weeks. The skin was examined in weeks 0 (baseline), 2, and 4. Before testing, the tested part was cleaned and dried, and skin testing was conducted after 30 min. The exclusion criteria included the following: skin disorders; liver diseases; kidney diseases; allergy to cosmetics, drugs or foods; pregnant and lactating women; people who had any cosmetic procedures (intense pulse light, medical peelings, or laser therapy) before 4 weeks of the study.

**TABLE 1:** Subject profile.

Group	Test group	Placebo group
Number (*n*)	20	20
Mean age (year)	49.1	41.5
Sex (F/M)	14/6	16/4

### 2.3 The Formula of *H. trueperi* TCI66207 Serum

The test group (*H. trueperi* TCI66207 serum) contained 1% TCI66207 lysate, 1,3‐butylene glycol, hydroxyacetophenone, hexanediol, xanthan gum, triethanolamine, and aqua. The placebo group (basic serum) contained 1,3‐butylene glycol, hydroxyacetophenone, hexanediol, xanthan gum, triethanolamine, and aqua.

### 2.4 Skin Measurement

Skin moisture (Corneometer CM825, Courage + Khazaka Electroni, Germany), brightness (Chroma Meter MM‐500, Minolta, Japan), and elasticity (Soft Plus Callegari 1930, Italy) of each subject's upper cheek were measured. The degree of improvement in skin moisture, brightness, and elasticity are positively correlated with the increase of measurement value.

VISIA Complexion Analysis (Canfield Scientific, Inc., Fairfield, NJ, USA) was employed to measure the skin spots and UV spots, textures, wrinkles, and pores of full face. DermaLab Series SkinLab Combo (Cortex) was employed to determine skin collagen density content. The instrument uses ultrasound to analyze the collagen density of the upper cheek. The results were presented as the mean value and the relative percentage (%) to the baseline.

### 2.5 Statistical Analysis

Data are presented as the mean ± standard error of mean (SEM) obtained from three repeats per sample. The comparison of measurement results for skin parameters among groups and between groups was analyzed by one‐way repeated measurement one‐way analysis of variance (ANOVA) and one‐way ANOVA, respectively, followed by Tukey's post hoc test through GraphPad Prism, as *p* < 0.05 was considered statistically significant.

## 3 Results and Discussion

### 3.1 Efficacy of *H. trueperi* TCI66207 Serum in Improving Skin Moisture, Brightness, and Elasticity

The study of microbes will reveal novel biochemicals, enzymes, and active ingredients useful to humans as industrial agents, food products, biopharmaceuticals, cosmetics, biofuels, and more. Marine microorganisms have anti‐oxidant, anti‐cancer, and anti‐microbial properties because of their produced bioactive compounds. Enormous research evidence has revealed that topical administration of live microorganisms and microorganism extracts can restore skin barrier and modulate cutaneous dysbiosis [12, 13, 14, 15]. This clinical study aims to explore whether *H. trueperi* improved skin aging; we used TCI66207, which is isolated from a 662 m deep Pacific Ocean in Hualien. As part of an evaluation of the efficacy of *H. trueperi* TCI66207 in skin, the effect of 4 weeks of applying *H. trueperi* TCI66207 serum on skin surface hydration, brightness, and elasticity was firstly examined.

After 4‐week *H. trueperi* TCI66207 intervention, the mean levels of skin moisture, brightness, and elasticity were increased by 8.9%, 2.3%, and 7.3%, respectively (*p* < 0.05) (Table 2). Recently, polysaccharide has been used in some industries like food, pharmacy, and cosmetics [16]. Most polysaccharides can be extracted from algae, plants, and bacteria [17]. The study showed that *H. trueperi* released EPS, a polysaccharide polymer containing sulfate and mannose, which had the effect of improving skin [18]. The EPS produced by lactic acid bacteria can effectively improve the moisture and elasticity of skin cells and prevent skin aging [19]. Moreover, *H. trueperi* TCI66207 lysate may reinforce the skin barrier and reduce trans‐epidermal water loss (TEWL). Skin brightness is associated with the skin tone and pigmentation [20]. In this study, the results of skin moisture, brightness, and elasticity were significantly different from the baseline results. Although the placebo also obviously ameliorated skin brightness and elasticity, *H. trueperi* TCI66207 still achieved better improving effects on the two indexes.

**TABLE 2:** Skin moisture, brightness, and elasticity after applying *H. trueperi* TCI66207 serum.

Item	Time‐point (W)	Test group (*n* = 20)				Placebo group (*n* = 20)			
		Mean value (SEM)	Improve rate (SEM) %	95% CI	*p*	Mean value (SEM)	Improve rate (SEM) %	95% CI	*p*
Moisture	0	65.5 (1.8)	100.0 (0.0)	0.0		69.0 (2.0)	100.0 (0.0)	0.0	
	2	66.7 (2.6)	101.7 (2.5)	4.9	0.497^a^, 0.577^b^	68.4 (2.3)	99.5 (2.5)	4.9	0.849
	4	71.5 (2.9)	108.9 (2.8)	5.5	0.004^a^, *, 0.302	71.1 (1.7)	104.1 (2.8)	5.5	0.154
Brightness	0	58.5 (0.6)	100.0 (0.0)	0.0		60.1 (0.5)	100.0 (0.0)	0.0	
	2	59.6 (0.6)	101.9 (0.5)	0.9	< 0.001^a^, *, 0.065^b^	60.6 (0.5)	100.8 (0.5)	0.9	0.100
	4	59.8 (0.5)	102.3 (0.6)	1.1	< 0.001^a^, *, 0.418	61.1 (0.5)	101.6 (0.5)	0.9	0.002
Elasticity	0	39.2 (1.4)	100.0 (0.0)	0.0		41.4 (1.2)	100.0 (0.0)	0.0	
	2	41.1 (1.3)	105.3 (1.2)	2.3	< 0.001^a^, *, 0.013^b^, *	42.0 (1.2)	101.4 (0.9)	1.7	0.120
	4	41.8 (1.2)	107.3 (1.4)	2.8	< 0.001^a^, *, 0.001^b^, *	42.2 (1.2)	102.0 (0.8)	1.6	0.025

*Note:* Sample size = 20; mean value ± standard error of the mean (SEM).

^a^ Compared the baseline (week 0) and the week 2 or week 4.

^b^ Compared the test group and the placebo group.

* Significantly different, *p* < 0.05.

### 3.2 Efficacy of *H. trueperi* TCI66207 Serum in Reducing Skin Spots, UV Spots, Textures, Wrinkles, and Pores

In light of the improvement of skin brightness, we postulated that the anti‐oxidants of *H. trueperi* TCI66207 may exert the depigmenting effect and inhibit tyrosinase activity considering its production of carotenoids. Pharmacological studies have shown that carotenoids may prevent the ultraviolet (UV) damage of skin by scavenging reactive oxygen species (ROS) in skin cells; ROS is responsible for melanogenesis on human melanocytes and fibroblasts [21]. The assumption could be partly verified by the results of skin spots and UV spots (Table 3). *H. trueperi* TCI66207 reduced the mean levels of skin spots and UV spots at week 4 by 11.0% and 7.6% (*p* < 0.05), respectively. Both skin indexes acquired remarkable amelioration after the study. As compared with the baseline results, the mean levels of skin textures, wrinkles, and pores of *H. trueperi* TCI66207 group at weeks 4 were decreased by 15.6% (*p* < 0.05), 27.2% (*p* < 0.05), and 4.6%, respectively (Table 3). Especially, the outcomes of skin textures and wrinkles demonstrated statistical significance.

**TABLE 3:** Skin spots, UV spots, texture, wrinkles, pores, and collagen density content after applying *H. trueperi* TCI66207 serum.

Item	Time‐point (W)	Test group (*n* = 20)				Placebo group (*n* = 20)			
		Mean value (SEM)	Improve rate (SEM) %	95% CI	*p*	Mean value (SEM)	Improve rate (SEM) %	95% CI	*p*
Spots	0	92.0 (7.5)	100.0 (0.0)	0.0		71.2 (7.3)	100.0 (0.0)	0.0	
	2	83.2 (6.8)	90.9 (3.2)	6.3	0.010^a^, *, 0.086^b^	68.9 (6.4)	99.8 (3.7)	3.7	0.956
	4	83.9 (8.2)	89.0 (2.6)	5.2	< 0.001^a^, *, 0.187^b^	67.1 (6.2)	96.9 (4.9)	4.9	0.536
UV spots	0	298.0 (15.0)	100.0 (0.0)	0.0		279.3 (13.5)	100.0 (0.0)	0.0	
	2	278.5 (16.4)	92.4 (1.9)	3.8	< 0.001^a^, *, 0.045^b^, *	275.5 (12.9)	99.2 (2.4)	4.7	0.735
	4	275.2 (15.8)	92.4 (2.1)	4.2	0.001^a^, *, 0.642^b^	260.7 (12.6)	94.4 (3.6)	7.1	0.140
Textures	0	395.8 (54.6)	100.0 (0.0)	0.0		234.7 (43.3)	100.0 (0.0)	0.0	
	2	358.3 (51.4)	90.5 (3.7)	7.2	0.018^a^, *, 0.506^b^	234.5 (44.1)	95.6 (6.4)	12.5	0.497
	4	334.8 (53.4)	84.4 (4.7)	9.3	0.003^a^, *, 0.436	228.6 (44.5)	90.7 (6.2)	12.1	0.148
Wrinkles	0	20.3 (2.0)	100.0 (0.0)	0.0		14.3 (2.0)	100.0 (0.0)	0.0	
	2	17.0 (1.8)	93.9 (9.1)	17.8	0.509^a^, 0.180^b^	13.8 (2.0)	109.1 (13.1)	25.6	0.495
	4	15.0 (2.2)	72.8 (7.3)	14.2	0.001^a^, *, 0.024*	14.5 (2.4)	111.6 (15.3)	30.1	0.457
Pores	0	807.6 (74.7)	100.0 (0.0)	0.0		722.3 (85.7)	100.0 (0.0)	0.0	
	2	753.3 (66.2)	95.2 (2.3)	4.6	0.054^a^, 0.250^b^	718.8 (86.5)	102.0 (5.1)	10.0	0.695
	4	740.7 (62.4)	95.4 (3.6)	7.1	0.221^a^, 0.452^b^	700.6 (80.2)	102.0 (8.6)	16.9	0.816
Collagen content	0	38.2 (4.0)	100.0 (0.0)	0.0		42.7 (3.4)	100.0 (0.0)	0.0	
	2	42.4 (4.2)	114.3 (4.5)	8.7	0.004^a^, *, 0.309^b^	45.4 (3.2)	110.5 (4.1)	8.0	0.019
	4	47.0 (4.0)	130.8 (8.4)	16.4	0.001^a^, *, 0.752^b^	52.5 (3.6)	127.7 (4.5)	8.9	< 0.001

*Note:* Sample size = 20; mean value ± standard error of the mean (SEM).

^a^ Compared the baseline (week 0) and the week 2 or week 4.

^b^ Compared the test group and the placebo group.

* Significantly different, *p* < 0.05.

**FIGURE 1:**
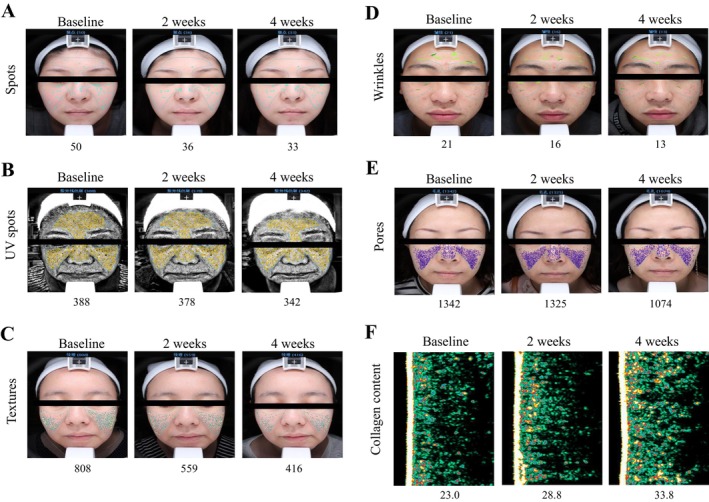
*H. trueperi* TCI66207 serum improved skin texture, wrinkles, pores, spots, UV spots, and collagen. After applying *H. trueperi* TCI66207 serum for 4 weeks, the subjects were examined based on (A) skin spots, (B) UV spots, (C) textures, (D) wrinkles, (E) pores by VISIA complexion analysis, and (F) collagen density content by DermaLab Series SkinLab Combo (Sample size = 20).

The EPS produced by the Arctic bacterium *Polaribacter* sp. SM1127 can provide a protective effect on human dermal fibroblasts against UV‐induced oxidative stress [22]. The Wnt signaling pathway was shown to be induced during aging in the muscle and in the skin [23]. The EPS isolated from a marine bacteria *Pantoea* sp. YU16‐S3 can facilitate cell migration in fibroblasts, induce rapid transition of cell cycle phases, and also activate macrophages through the Wnt/β‐catenin pathway [24]. In addition, the study indicated that *H. trueperi* can produce astaxanthin, which is a carotenoid with potent anti‐oxidant and anti‐inflammatory activity [25]. Astaxanthin treatment suppressed ultraviolet B (UVB)‐induced inflammatory cytokine secretion in keratinocytes by scavenging ROS [26]. ROS is responsible for melanogenesis on human melanocytes and fibroblasts [27]. Moreover, a clinical trial also indicated that oral astaxanthin can improve skin wrinkles. In this study, Figure 1A–F shows that skin images included spots, UV spots, textures, wrinkles, pores, and collagen density content. After 4 weeks of *H. trueperi* TCI66207 serum consumption, the moisture, brightness, elasticity, spots, UV spots, textures, wrinkles, and pores of skin were slightly better than those at week 0.

### 3.3 Efficacy of *H. trueperi* TCI66207 Serum in Promoting Skin Collagen Density

Carotenoids can absorb the UV radiation and counteract the rise of oxidative stress, which involves the expression of matrix metallopeptidases (MMPs) and elastases in keratinocytes and fibroblasts [28, 29]. The up‐regulation of the extracellular matrix (ECM) proteolytic enzymes leads to the destruction of the ECM structure [26]. *H. trueperi* TCI66207 promoted a 30.8% (*p* < 0.05) increase in collagen density after the study, although there was a significant improvement with placebo (Table 3). We speculate that the EPS secreted by *H. trueperi* TCI66207 can promote collagen formation. The increase in EPS can also stimulate the collagen production [30]. In addition, *H. trueperi* can produce carotenoid, which can inhibit MMPs in keratinocytes and fibroblasts [31]. *H. trueperi* TCI66207 may not only suppress the expression of proteolytic enzymes but reinforce the skin structure and firmness.

## 4 Conclusion

This study revealed a successful demonstration of *H. trueperi* TCI66207 for skin health as evidenced by the remarkable and comprehensive improvement of skin parameters. Although the underlying mechanisms require further investigation, the preliminary results indicate the potential of *H. trueperi* TCI66207 for retarding the process of skin aging. We believe that our efforts here can extend the development of marine microorganisms in pharmaceutical and cosmetic realms.

## Author Contributions

C.‐H.L. analyzed and wrote the paper; L.‐P.C. wrote and edited the paper; D.‐L.C. and Y.‐P.T. developed and provided the supplement.

## Ethics Statement

This clinical research was approved by the ethics committee of the Antai Medical Care Corporation Antai Tian‐Sheng Memorial Hospital (TSMH‐IRB 17‐095‐A), and the study was conducted at Chia Nan University of Pharmacy and Technology. All recruited subjects returned written consent forms.

## Conflicts of Interest

The authors declare no conflicts of interest.

## Data Availability

The data used to support the findings of this study are available from the corresponding author upon request.
